# Genome Sequence of a Lethal Strain of Xylem-Invading *Verticillium nonalfalfae*

**DOI:** 10.1128/genomeA.01458-17

**Published:** 2018-01-11

**Authors:** Jernej Jakše, Vid Jelen, Sebastjan Radišek, Ronnie de Jonge, Stanislav Mandelc, Aljaž Majer, Tomaž Curk, Blaž Zupan, Bart P. H. J. Thomma, Branka Javornik

**Affiliations:** a Centre for Plant Biotechnology and Breeding, Department of Agronomy, Biotechnical Faculty, University of Ljubljana, Ljubljana, Slovenia; b Slovenian Institute of Hop Research and Brewing, Žalec, Slovenia; c Bioinformatics & Systems Biology, Ghent University, Ghent, Belgium; d Plant-Microbe Interactions Department, Utrecht University, Utrecht, The Netherlands; e Laboratory of Bioinformatics, Faculty of Computer and Information Sciences, University of Ljubljana, Ljubljana, Slovenia; f Laboratory of Phytopathology, Department of Plant Sciences, Wageningen University and Research, Wageningen, The Netherlands

## Abstract

*Verticillium nonalfalfae*, a soilborne vascular phytopathogenic fungus, causes wilt disease in several crop species. Of great concern are outbreaks of highly aggressive *V. nonalfalfae* strains, which cause a devastating wilt disease in European hops. We report here the genome sequence and annotation of *V. nonalfalfae* strain T2, providing genomic information that will allow better understanding of the molecular mechanisms underlying the development of highly aggressive strains.

## GENOME ANNOUNCEMENT

*Verticillium nonalfalfae* isolates from hops express two types of aggressiveness, appearing as a mild or lethal form of the wilt disease. This disease is characterized by plant stunting and wilting, vascular browning, foliar chlorosis and necrosis, and, in the case of the lethal form, rapid plant withering and dieback (1). By comparative genomics of mild and lethal *V. nonalfalfae* hop isolates (BioProject no. PRJNA283258), we anticipated the dissection of the pathotype genomes to provide an insight into their genomic structure. This in turn might explain the increased virulence of the lethal strain, enable detection of virulence-associated factors, and elucidate the pathogenicity in *Verticillium* spp.

DNA from protoplasts prepared from *V. nonalfalfae* conidia that germinated overnight (2) was isolated following the cetyltrimethylammonium bromide (CTAB) protocol (3). Whole-genome sequencing was carried out by IGA Technology Services (Udine, Italy) and the Beijing Genomics Institute (BGI, Hong Kong) using Illumina Genome Analyzer IIx and HiSeq 2500 systems with a paired-end setup. For the reference genome assembly (strain T2), four libraries of different insert sizes (5,000-bp mate paired-end and 1,000-bp, 500- to 600-bp, and 370-bp paired-end inserts) were sequenced (BioSample no. SAMN03610599). In total, 98 million raw reads were generated, representing a 390× sequencing depth. Reference genome assembly was performed using the CLC Genomics Server (ver. 5.0.1) *de novo* assembly tool (Qiagen, USA). Sequencing data were assembled in 409 scaffolds with a G+C content of 55.35% and a total assembly size of 34.18 Mb. The *N*_50_ length was 292,274 bp, the largest scaffold was 1,095,096 bp, and 50% of the assembly was represented by 34 scaffolds. There were 1,740 unknown nucleotides (nt) per 100 kb. In addition, RNA sequencing of mild and lethal *V. nonalfalfae* pathotypes from Slovenia was performed (Sequence Read Archive accession no. SRX1020679 and SRX1020629) using total RNA [enriched for the poly(A) mRNA fraction] isolated from fungal mycelia grown in xylem-simulating medium (4). For gene annotation, we followed the *V. dahliae* annotation pipeline (5), in which various evidences of genic regions are combined into a final gene model, as follows: i) Cufflinks (6) identification of transcripts with transcriptome sequencing (RNA-seq) data and PASA assembly of transcripts (7), ii) protein alignments of 15 fungal species obtained by AAT (8) and Exonerate software (9), and iii) *ab initio* prediction using GeneMark-ES (10) and Augustus (11) predictions. EVidenceModeler software (7) was then used to obtain gene models. The finalized genome annotation includes 9,269 protein coding genes. Most of the genes have a coding sequence (CDS) length in the range of 1,000 to 2,000 bp, with the majority being <5,000 nucleotides (nt). Most genes have <5 exons, with a prevalence of 2 exons. The CEGMA pipeline (12) identified 445 of the 458 clusters of orthologous groups (COGs) to be present (completeness, 97.2%).

Additionally, lower-coverage sequencing (paired-end) of 2 lethal and 3 mild strains was performed and accumulated from 9.6 to 43.9 million reads (BioSample no. SAMN03612289, SAMN03611609, SAMN03610662, SAMN03612288, and SAMN03612257). Comparative genomics of mild and lethal *V. nonalfalfae* pathotypes revealed a 0.547-kb lethal pathotype-specific region, which is absent in mild pathotypes.

### Accession number(s).

This whole-genome shotgun project has been deposited at DDBJ/EMBL/GenBank under the accession no. PDEZ00000000. The version described in this paper is the first version, PDEZ01000000.
